# Negative Perception of the COVID-19 Pandemic Is Dropping: Evidence From Twitter Posts

**DOI:** 10.3389/fpsyg.2021.737882

**Published:** 2021-09-28

**Authors:** Alessandro N. Vargas, Alexander Maier, Marcos B. R. Vallim, Juan M. Banda, Victor M. Preciado

**Affiliations:** ^1^Electronics Department, UTFPR, Universidade Tecnológica Federal do Paraná, Cornelio Procópio-PR, Brazil; ^2^Department of Psychology, College of Arts and Science, Vanderbilt Vision Research Center, Vanderbilt University, Nashville, TN, United States; ^3^Department of Computer Science, College of Arts and Sciences, Georgia State University, Atlanta, GA, United States; ^4^Department of Electrical and Systems Engineering, University of Pennsylvania, Philadelphia, PA, United States

**Keywords:** negative perception, psychophysical numbing, Twitter, COVID-19, vaccine

## Abstract

The COVID-19 pandemic hit hard society, strongly affecting the emotions of the people and wellbeing. It is difficult to measure how the pandemic has affected the sentiment of the people, not to mention how people responded to the dramatic events that took place during the pandemic. This study contributes to this discussion by showing that the negative perception of the people of the COVID-19 pandemic is dropping. By negative perception, we mean the number of negative words the users of Twitter, a social media platform, employ in their online posts. Seen as aggregate, Twitter users are using less and less negative words as the pandemic evolves. The conclusion that the negative perception is dropping comes from a careful analysis we made in the contents of the *COVID-19 Twitter chatter dataset*, a comprehensive database accounting for more than 1 billion posts generated during the pandemic. We explore why the negativity of the people decreases, making connections with psychological traits such as psychophysical numbing, reappraisal, suppression, and resilience. In particular, we show that the negative perception decreased intensively when the vaccination campaign started in the USA, Canada, and the UK and has remained to decrease steadily since then. This finding led us to conclude that vaccination plays a key role in dropping the negativity of the people, thus promoting their psychological wellbeing.

## 1. Introduction

Social media has become virtually ubiquitous, driving the flow of information across the globe and governing how most of us receive and share information (Willnat and Weaver, 2018). The impact of social media on individuals is significant. For instance, one study reports that around 62.4% of the Vietnamese adults rely on social media as a source of news (Huynh et al., 2020), and around 67% of the U.S. adults at least occasionally get news on social media (Matsa and Shearer, 2018). Most Facebook users spend one or more hours per day on its platform (Ernala et al., 2020, Figure 3), let alone the hours spent on other platforms like Instagram, WhatsApp, and Snapchat (e.g., Verbeij et al., 2021). Twitter, another important social media platform, has been used intensively by young adults (Antonakaki et al., 2021). For instance, one study has found that more than 80% of the individuals in a group of undergraduate students spend two or more hours per day on Twitter (Bicen and Cavus, 2012).

Intense activity on social media elicits some negative reactions. For instance, people using various social media platforms have substantially higher levels of both depression and anxiety when compared to those who use two or less social media platforms (Primack et al., 2017), impacting strongly teenagers (Woods and Scott, 2016). One study shows that 20% of college students in a population were addicted to social media (Allahverdi, 2021). Other studies confirm that being intensively exposed to news and social media negatively affects the mental health of an individual (Brunborg and Burdzovic Andreas, 2019; Brailovskaia et al., 2021; Geirdal et al., 2021). Also, diversity of thought can disappear and studies report that social media users engage in similarly thinking groups, framing, and reinforcing a shared narrative, a psychological phenomenon called *echo chambers* (Cinelli et al., 2021; Lavorgna and Myles, 2021; Mosleh et al., 2021). Despite the psychological risks (Primack et al., 2017; Allahverdi, 2021), people have become used not only to spending long hours on social media but also to expressing sentiment and opinions therein (De Choudhury et al., 2016; Guntuku et al., 2017).

Among the many social media platforms available, one has attracted increasing attention over the last few years: Twitter. Twitter is a social media platform that allows users to share messages containing up to 280 characters (Saleh et al., 2021). A survey conducted in 2014 found that 23% of adults online were using Twitter (Cavazos-Rehg et al., 2016). For this reason, journalists have used Twitter as a mainstream media for monitoring news and communicating with their audiences (Willnat and Weaver, 2018). Yet Twitter has been used also to spread misinformation and hoaxes (Balestrucci et al., 2021).

Twitter has caught the attention of researchers as well, and researchers have seen Twitter posts as an invaluable source of information about the thoughts and feelings of people (e.g., Cavazos-Rehg et al., 2016; Saif et al., 2016; Jaidka et al., 2020; Antonakaki et al., 2021; Mosleh et al., 2021). A Twitter post, called simply as a *tweet*, carries a piece of text that can be analyzed to determine the user's depressive thoughts (Cavazos-Rehg et al., 2016), sleep disorders (McIver et al., 2015), cognitive behavior (Mosleh et al., 2021), and wellbeing of the users (Saif et al., 2016; Jaidka et al., 2020). More recently, during the COVID-19 pandemic, researchers have used tweets to assess how the wellbeing of the people has been affected, as detailed next.

The COVID-19 pandemic hit nations hard, forcing governments and individuals to take unprecedented measures to contain the spread of the disease (e.g., Henríquez et al., 2020; Giuntella et al., 2021). Several studies confirm the pandemic has worsened the overall mental health (see Huang, 2020; Marques de Miranda et al., 2020; Achterberg et al., 2021; Daly and Robinson, 2021b; de Figueiredo et al., 2021; Varma et al., 2021 for a brief account). The way in which the mental health has been impacted comes from distress factors like fear of contracting the disease and concerns about the health, unemployment, subsistence, stay-at-home orders, and prolonged social isolation of the relatives (Pietrabissa and Simpson, 2020; Daly and Robinson, 2021b; Lavigne-Cerván et al., 2021; Varma et al., 2021).

**Figure 1:**
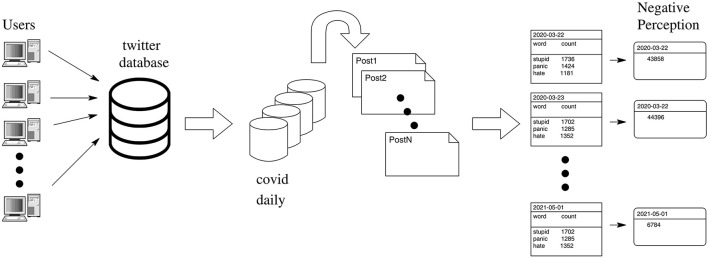
Flow of the Twitter data processing. Users feed the Twitter database with pieces of text (i.e., posts), and an algorithm extracts from this database only the posts related to the COVID-19 pandemic (e.g., Banda et al., 2021). The COVID-related posts are then processed on a daily basis to generate tables containing the negative words and the number of their ocurrences. The negative perception represents the sum of all ocurrences taken daily from these tables.

Researchers have monitored Twitter posts to extract information about the wellbeing of the people during the pandemic. In the beginning of the pandemic, researchers have used Twitter to classify the most discussed topics (Su et al., 2021) and hashtags (e.g., #stayhome) (Petersen and Gerken, 2021), as well as to assess whether Twitter users supported social distancing (Saleh et al., 2021)—note that *fear* was found in 20% of the corresponding posts (Saleh et al., 2021). Another study has investigated the sentiment of the Twitter users (Dyer and Kolic, 2020), showing that the negative sentiment surged in the beginning of the pandemic. The authors of Garcia and Berton (2021) have found that the negative sentiment toward the pandemic increased from January 19 to March 3, 2020, coinciding with that finding from Dyer and Kolic (2020). Another study analyzed more Twitter data to conclude that negative feelings increased just after the beginning of the pandemic (Wicke and Bolognesi, 2021). These contributions confirm that negativity increased at the beginning of the pandemic. Although we acknowledge that the negative sentiment increased when the pandemic started, here we report that the negative sentiment is dropping steadily. As detailed below, the data now show that the negativity decreased almost linearly during the vaccination campaign (see section 3.4). As the main finding of this study shows, vaccination, thus, might induce an important psychological benefit—reducing the negativity of the people. The main contribution of this study is to determine the negative perception of Twitter users during the COVID-19 pandemic, see Figure 1.

By negative perception, we mean the number of negative words contained in a collection of tweets. While the English lexicon of negative words is large, accounting for more than 3,000 words (e.g., Clore et al., 1987; Mohammad and Turney, 2013; Hutto and Gilbert, 2014, we selected from this lexicon only few words that carry a strong negative connotation. We did so because some words may be either negative or positive, depending on the context (Poria et al., 2020; Mohammad, 2021). The resulting list of negative words is in Table 1.

**Table 1:** List of negative words.

**Negative words**
panic, fear, sad, mental, mind, sorry, shame, hate, hell, violence,
bad, feel, feeling, shit, worst, worse, blame, lonely, horrible,
chaos, mad, ruined, anxiety, stress, stressed, phobia, abuse,
shame, hurt, disorder, loneliness, turmoil, anger, horror,
rage, fate, nervous, restless, depression, grief, worry, stupid
worried, angst, depressed, suicide, suffer, suffering,
uncertainty, uneasy, sadness, afraid, alone, suicidal, mood,
tension, anxious, desperate, dismal, exhausted, insecure,
distress, distressed, frustration, disgusting, boredom, bored,
insane, stupidity, bullshit, uncertain, displeased, upset, outrage,
uncomfortable, melancholy, overwhelmed, pessimistic, unhappy,
ass, damn, covidiot, terrify, terrified, terrifying, distrust,
scare, scared, scaring, dread, failed, failed, failure, crisis,
fuck, fucking, miserable, regret, shock, shocked, rape, mess,
negligence, betrayed, cry, crying, idiot, idiots, selfish, upheaval.

**Figure 2:**
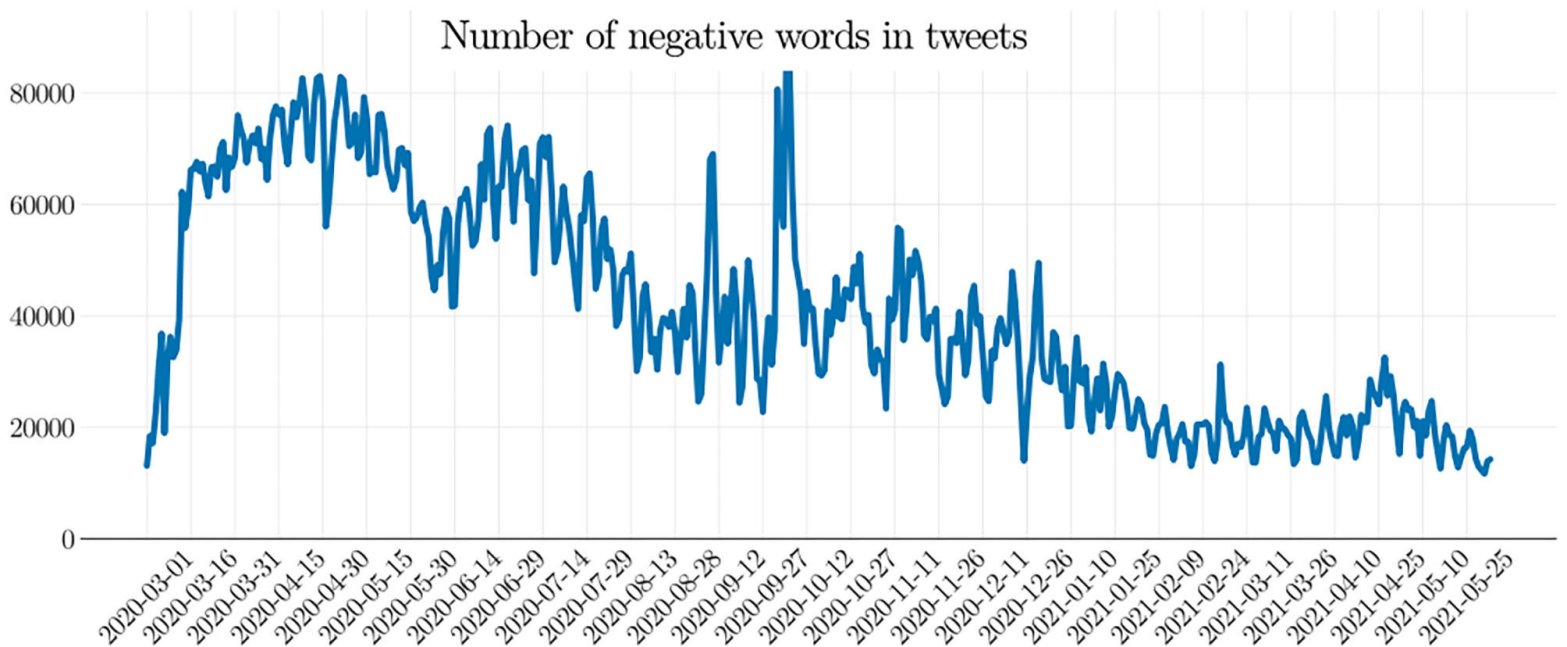
Occurrence of negative words found in COVID-19-related Twitter posts. As the COVID-19 pandemic evolves, the number of negative words decreases. This fact suggests that the overall negative perception about the COVID-19 pandemic be diminishing.

To measure negative perception, we analyzed over 150 million tweets that contained words related to the COVID-19 pandemic. These tweets were collected from March 1, 2020, to June 2, 2021, and were archived in Banda et al. (2021). Having calculated the negative perception on a daily basis, we see negative perception toward the pandemic is dropping, and this fact represents the main finding of this study (see Figure 2 for a pictorial illustration).

While there exist many reasons why negative perception is dropping, as discussed in section 3, we find a strong correspondence between the diminished negative perception and the vaccination campaign in the USA (see section 3). Even though many individuals oppose to the vaccination (Germani and Biller-Andorno, 2021), our finding indicates that the vaccination has decreased the negativity of the Twitter user.

The takeaway message from this study is as follows. The negativity of the people has dropped. In particular, the negativity of the people declined almost linearly as the vaccination rose exponentially, suggesting slow emotional adaptation to a rapidly evolving situation. For this reason, it seems reasonable to affirm that the vaccination campaign has played a crucial role in decreasing the negativity of the people. In addition to the vaccination campaign and its perception, this study explores several psychological traits that could have affected the negativity of the people during the pandemic. These findings help us understand why and how the negative perception of the people has evolved during the pandemic.

## 2. Methods and Procedures

Figure 1 summarizes the methods and procedures developed in this study. The Twitter database we used in our analysis is the “*COVID-19 Twitter chatter dataset”* freely available in Banda et al. (2021). It comprises the most comprehensive COVID-19 Twitter dataset available on the internet, reaching more than 1 billion tweets collected during the pandemic. All the Twitter posts from this database are related to the COVID-19 pandemic. A particular feature of this database is that it contains only original tweets, that is, it is free of *retweets* (a retweet is a re-post of an original tweet), an advantage of the COVID-19 Twitter dataset (Banda et al., 2021).

An ID number uniquely identifies each Twitter post. According to the policy of the Twitter, only the ID number can be shared. To gain access to the full content, an individual has to gain permission from the Twitter company and abide by a confidentiality contract. Only after granted permission can an individual use the ID number to download the full content of a post, following a process called “*hydration.”* To *hydrate* a tweet, the authors of Banda et al. (2021) recommend using the “*Social Media Mining Toolkit”* (Tekumalla Ramya, 2020). We used this toolkit to gain full access to the contents of the COVID-19 Twitter dataset (Banda et al., 2021).

Since this study focuses on the negative perception of the pandemics in the English-speaking community, we consider only tweets written in English. Extracting only the tweets in English became possible because Twitter uses an algorithm that automatically detects the idiom of each posted message (a post in English carries the attribute “lang = en”). Around 120 million tweets in English were analyzed, representing posts collected from March 1, 2020, to June 2, 2021.

**Remark 1**. *Institutional review board approval is unnecessary for this study because all the data used here are publicly available and were processed in aggregate, according to the Twitter policies (e.g.*, https://twitter.com).

### 2.1. Definition of Negative Perception in Tweets

According to the literature, people perceive negative events more intensively than positive ones, a phenomenon called *negativity bias* (Rozin and Royzman, 2001). When comparing events of the same nature (i.e., receiving or losing something), the bad event brings about much stronger psychological effects than the good event (Baumeister et al., 2001; Rozin and Royzman, 2001). The same bias applies to stimuli, and even early infants pay more attention to negative than to positive stimuli (Vaish et al., 2008). Not surprisingly, the negativity bias drives how humans see the world (Soroka et al., 2019).

Some researchers have argued that all emotions are valenced, i.e., emotions are either positive or negative, but never neutral (Ortony et al., 1990). A form of expressing emotion is language, mostly associated with sentiment and perception (Berry et al., 1997; Lindquist, 2017). Being a key element in language processing, writing determines how others perceive an the feelings of an individual (Ortony et al., 1990, p. 15). Because writing and individual words carry a certain level of emotion, researchers have attempted to characterize the sentiment of an individual through word analysis (Ortony et al., 1990; Taboada et al., 2011; Liu, 2015). The idea is that each word has a definite emotion. For instance, *happiness, joy*, and *benevolent* bring a positive sentiment, while *hate, fear*, and *anxiety* bring a negative sentiment.

Assessing emotion or sentiment from a piece of writing comprises a research area known as *sentiment analysis* (Cambria et al., 2013; Liu, 2015; Nazir et al., 2020). Research study in this area focuses on methods that extract sentiment automatically from a text. Using algorithms to accomplish that task, researchers have driven the investigations toward two fields: *lexicon analysis* and *machine learning*. Both fields depend on human intervention, as detailed next.

Lexicon analysis hinges upon a list of words in the form of a table. Each entry contains a word and its sentiment score. To illustrate what this means, let us discuss the contribution in De Choudhury et al. (2016). Individuals have been asked to rank their perception about the positivity and negativity of words in a list, issuing a number from −5 (most negative) to 5 (most positive), see Taboada et al. (2011). Gathering the data, the authors have created a table containing around 5,000 entries (De Choudhury et al., 2016). Among them, we can cite—for the sake of illustration—the words *agony* (−4), *anxiety* (−1), and *inspiration* (+3). Yet the authors mention that they observed a high number of disagreements, i.e., a word is seen as positive for some individuals and negative for others.

Other sentiment tables exist, see, for instance, a sentiment table containing 1,034 words in Stevenson et al. (2007), other containing around 6,800 words in Liu (2015), other containing around 11,000 words in Stone (1997), and another containing around 14,000 words in Mohammad and Turney (2013). Available in the form of a commercial product, yet another sentiment table was created with around 6,400 words (Tausczik and Pennebaker, 2010). More recently, these sentiment databases have been expanded and redefined, adding a combination of consecutive words and expressions. This allowed researchers to extract sentiment not only from words but also from phrases or pieces of text (Cambria, 2016; Saif et al., 2016; Nazir et al., 2020). Even though we can see significant progress in constructing sentiment tables, how to choose a sentiment score is still debatable.

The machine learning approach has been used to build sentiment score numbers in a plethora of ways. For instance, the authors of Hutto and Gilbert (2014) have combined the sentiment scores from Stone (1997), Liu (2015), and Tausczik and Pennebaker (2010), in a way that accounted for certain grammatical and syntactical conventions. After finishing this task, which involved personal judment, the authors have issued a large sentiment database, which was named *VADER*, see Hutto and Gilbert (2014). In addition to being freely available, VADER has attained certain popularity within the academic community. For instance, researchers have used VADER to determine the sentiment of students toward teachers (Newman and Joyner, 2018), to find sentiments toward bitcoin and cryptocurrency on Twitter (Cavalli and Amoretti, 2021), and to extract sentiments from e-mails (Borg and Boldt, 2020), and from Amazon reviews (Dey et al., 2018).

Regarding natural language processing (NLP), a tool that has been widely used is the so-called *BERT* (Devlin et al., 2018). The creators of BERT, members of a research team working for Google, mention that BERT incorporates a training database of writings with more than a billion words (see Devlin et al., 2018), a feature that has helped BERT reach success in more than 90% of the classification tasks (e.g., Alaparthi and Mishra, 2021). Note, however, that even well-trained judges do not agree with each other in rating setiment from personal stories (Tausczik and Pennebaker, 2010, p. 26). Judges tend to perform better than an algorithm when the task is detecting depression (Ziemer and Korkmaz, 2017). Although these investigations taken together indicate that researchers have gone through highly technical, complicated methods when extracting sentiment from words and phrases, understanding the full complexity of those algorithms and their score numbers prevents their widespread use.

Instead of relying on those algorithms, here we follow the traditional procedure of counting the number of negative words. To us, a negative word means a word that almost definitively brings about a negative perception of reality, like *hate, fear, anxiety*, and others (see Table 1). Before discussing how those negative words were chosen, we note that counting the number of negative words in a post is much less computationally intensive than computing a sentiment score through machine learning algorithms—a clear advantage of our approach.

The negative words considered in this study were selected according to the following two procedures. First, we selected manually words we consider unambiguously negative from the lists of the 1,000 most common words published daily at Banda et al. (2021). Next, we hand-picked negative words from both the VADER lexicon (e.g., Hutto and Gilbert, 2014) and the NRC emotion lexicon (e.g., Mohammad and Turney, 2013), and we considered them after checking that they appeared in tweets—the resulting list of negative words is in Table 1. It is worth noting that many negative words were dismissed because they were either potentially ambiguous or their ocurrences in the tweets were statistically insignificant.

**Definition 1**. *(Negative perception in tweets). The negative perception in tweets is the number of negative words found in the COVID-19 Twitter chatter dataset (Banda et al.*, *2021**). The negative perception in tweets is calculated daily*.

All the negative words were searched within the COVID-19 Twitter chatter dataset on a daily basis, and the corresponding negative perception *N*(*d*) on the *d*-th day was recorded and used to calculate the negative frequency index, which equals


(1) $$\begin{array}{r} I\left(d\right)=\frac{N\left(d\right)}{T\left(d\right)}, \end{array}$$

where *T*(*d*) represents the total number of tweets on the *d*-th day.

## 3. Results and Discussion

The number of negative words in the COVID-19 Twitter chatter dataset is shown in Figure 2. As can be seen, there exists a trend of diminishing the number of negative words as the pandemic evolves. This is a clear indication that the overall negative perception toward the COVID-19 pandemic is dropping. A number of theories can be sought to explain why this phenomenon is happening. We explore some of them in the following.

**Figure 3:**
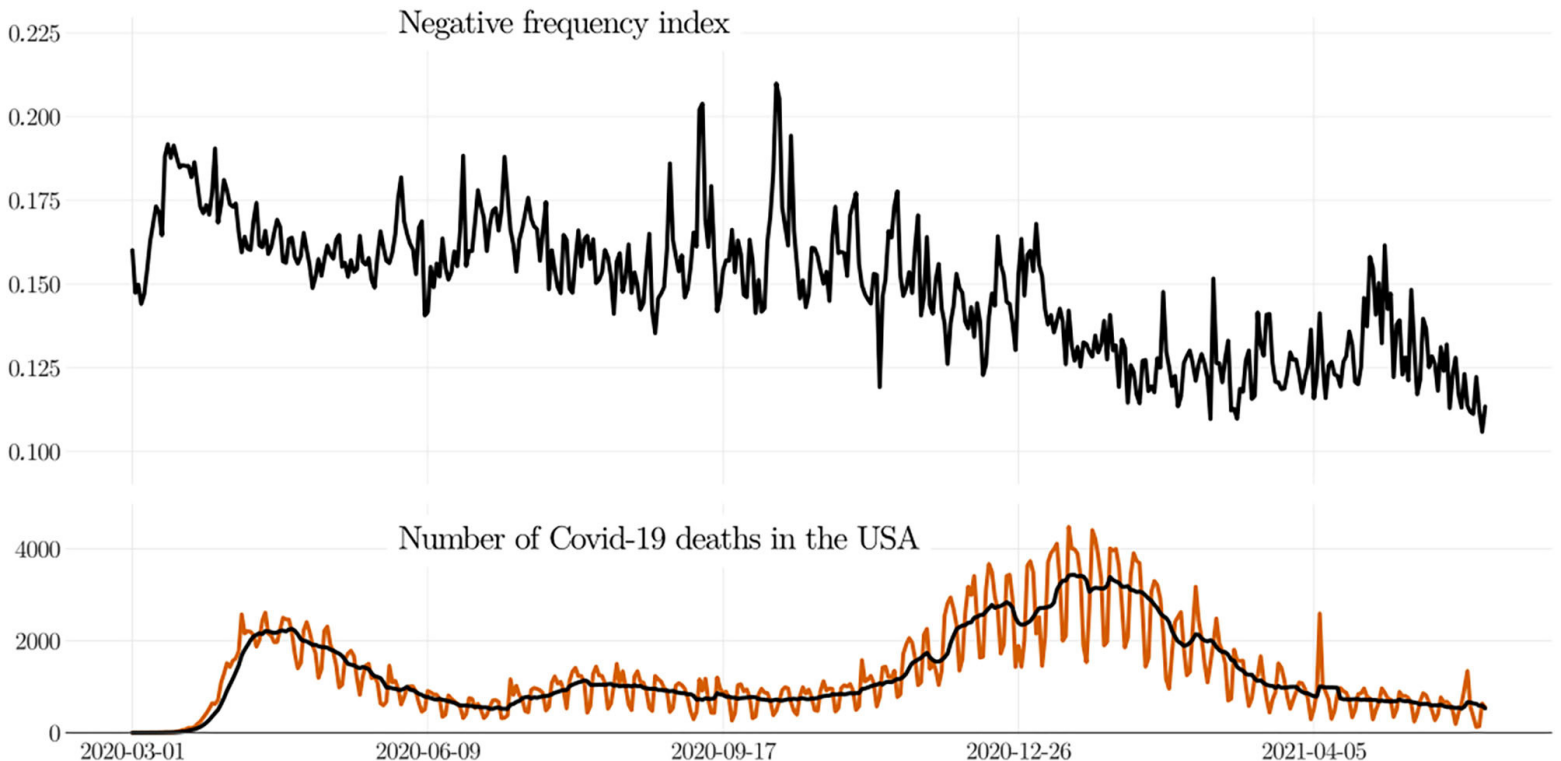
Negative frequency index (negative perception in tweets) and the statics of COVID-19 deaths in the USA (death data from Hasell et al., 2020). Although the USA accounts for around 42.5% of all tweets (e.g., Garcia and Berton, 2021), the negativity decreased when the number of deaths skyrocket in the end of 2020, a paradox that seems to be driven by *psychophysical numbing*.

**Remark 2**. *When the number of negative words is normalized by the number of tweets per day, we obtain the negative frequency index, see (1). The evolution of this index is shown in* *Figure 3**. As can be seen, the negative frequency index is dropping, following a trend similar to that observed in* *Figure 2**. The curves from* *Figures 2*, *3* *allow us to conclude that the the negativity of the people toward the pandemic is diminishing*.

**Remark 3**. *President Donald Trump's opinions had a large impact on Twitter. For instance, one study found that Donald Trump's tweets containing negative sentiment were responsible for increasing volatility in Bitcoin prices (Huynh, 2021). We observe the negative perception in tweets shows spikes from October 2 to October 6, 2020, probably related to the news released on October 2 that President Donald Trump tested positive for COVID. After this event, the negative perception in tweets started dropping steadily*.

### 3.1. Psychophysical Numbing

The term *psychophysical numbing* is used in the literature to refer to a striking human condition: Individuals become less worried when the population suffering increases (Fetherstonhaugh et al., 1997; Friedrich et al., 1999; Slovic and Västfjäll, 2015; Bhatia et al., 2021; Maier, 2021). It implies that people become insensitive or “numbed” to one death when it happens in the middle of many deaths (Friedrich et al., 1999, p. 278).

The psychophysical numbing drives the sentiment of compassion toward helping others. For instance, one study suggests that the motivation of a person for helping people decrease when the number of people in need increases (Butts et al., 2019). Another study indicates that individuals tend to respond more strongly toward the suffering of one individual than to the suffering of a group (Cameron and Payne, 2011). In another study, after analyzing published news and social media posts, researchers have found evidence of the psychophysical numbing: Emotions of fear and anger were more common in texts mentioning fewer deaths (Bhatia et al., 2021). More recently, one study recalls the human condition in which an individual tends to emphasize more intensively smaller deviations in size while underestimating the larger ones, connecting this human trait with the distorted perception of the COVID-19 data (Maier, 2021). These investigations point out the common perception that individuals cannot comprehend the human suffering and losses of life as the corresponding numbers increase (Slovic and Västfjäll, 2015).

### 3.2. Reappraisal and Suppression

*Reappraisal* denotes a condition in which an individual changes the way a situation is construed in order to decrease its emotional impact (Gross, 2002; Nezlek and Kuppens, 2008; English et al., 2017). When feeling a negative emotion, a person doing the reappraisal construes an interpretation of the stressor event to decrease the experienced negative emotion (Gross, 2002; Nezlek and Kuppens, 2008; English et al., 2017). *Suppression* means a condition in which people suppress expressing to others their inner feelings (Gross, 2002; Nezlek and Kuppens, 2008; Brockman et al., 2017). Suppression represents the conscious decision of an individual to suppress thinking about the situation and get it out of their awareness. While avoiding expressing their negative emotion to others, individuals comply with the perceived social pressure that sees a negative emotion as bad (Bastian et al., 2017). Both conditions have been intensively studied over the last decades, including from the neurological viewpoint (Goldin et al., 2008; Arias et al., 2020).

### 3.3. Resilience

*Resilience* is a term used to denote the ability of an individual to overcome significant life adversity (Ungar and Theron, 2020; Masten et al., 2021). Resilience of an individual depends not only on the adversity itself but also on the culture and context in which the individual is immersed. It is well-known that the resilience of an individual is influenced by distinct factors, such as his or her level of education, age, employment, family support, and social insertion (Ungar and Theron, 2020; Masten et al., 2021).

One study reports that resilience is a recovery process, i.e., the adversity declines in the perception of an individual while improvements take place (Infurna and Luthar, 2018). The recovery is obtained when the wellbeing of an individual returns to a level close to the one they had before the adversity took place (Infurna and Luthar, 2018). For instance, one study reports that the population of Galveston Bay, Texas, USA, had shown resilience when Hurricane Ike hit them—most people showing psychiatric disorders due to this natural disaster recovered soon afterward (Pietrzak et al., 2012).

### 3.4. Discussion About the Perception of the COVID-19 Pandemic

As Figure 2 indicates, the negative perception of the COVID-19 pandemic is dropping among Twitter users. The most likely factors behind that trend are discussed next.

One study has shown that the level of distress in the USA climbed in March 2020 and reached a peak in April 2020, yet it returned to the March level in June 2020 (see Daly and Robinson, 2021b, Figure 1). This coincides with the negative perception in tweets. Note in Figure 2 that the negative perception in tweets reached a peak at the end of March and decreased to the lowest value in June, in accordance with the distress level in the USA (Daly and Robinson, 2021b). This comparison is valid only for the beginning of the pandemic because the study in Daly and Robinson (2021b) does not contain data after June 2020.

Another study analyzed the data for the population in the USA suffering from anxiety, data collected from January 2019 (long before the pandemic) to January 2021 (see Daly and Robinson, 2021a). The data show that anxiety afflicted less than 8% of the population before the pandemic, but it jumped to a peak of more than 20% at the beginning of April 2020. The peak of anxiety registered in April 2020, which is consistent with the highest unemployment rate recorded in the USA since the Great Depression (Couch et al., 2020). After April 2020, the anxiety level decreased and reached 12% in May 2020, remaining at this value until January 2021 (e.g., Daly and Robinson, 2021a). That the anxiety level remained stable from May 2020 to January 2021 disagrees with the diminishing negative perception in tweets. What produces this dichotomy is discussed next.

We believe that the COVID-19 pandemic and its deadly consequences have ignited on us psychological defenses, such as *psychophysical numbing, reappraisal, suppression*, and *resilience*. *Resilience*, for instance, was the most common psychological trait found in a population during the pandemic, as one study reports (Valiente et al., 2021). *Reappraisal* has found support in one study that emphasizes the necessity of incorporating positive psychology practices to help individuals cope with the COVID-19 pandemic (Waters et al., 2021). One study has analyzed the emotions of the people during the lockdown and how they rebalanced their positive systems (Mariani et al., 2021), a psychological trait associated with *reappraisal*. *Suppression* is the key mechanism behind the attempts of the people to remove from their consciousness thoughts related to death during the pandemic (Pyszczynski et al., 2021, p. 177). *Psychophysical numbing* is a complex trait also studied during the pandemic (Dyer and Kolic, 2020), as detailed next.

As most of us would probably intuit, increasing COVID deaths must increase the negative perception of the people. However, evidence shows the opposite. Figure 3 depicts both the negative frequency index and the death toll in the USA (death data from Hasell et al., 2020). As can be seen, the negative frequency index varies within the interval [0.15, 0.18] from the beginning of the pandemic until the end of November 2020. After that period, the negative frequency index started decreasing, even though the death toll in the USA started increasing sharply, reaching an astounding number of more than four thousand deaths per day.

What the data reveal is that the increase in COVID-19 deaths in the USA, after November 2020, coincides with a pronounced decrease in negative perception—a paradox. Curiously, a similar paradox emerged when the pandemic started in the USA: The psychological distress index rapidly diminished just after few weeks while the number of deaths remained increasing (Daly and Robinson, 2021b). This paradox in the in the perception of the people seems to agree with the well-known psychological trait called *psychophysical numbing*, which copes with the quote “*the more who die, the less we care,”* see Slovic and Västfjäll (2015); Dyer and Kolic (2020); Bhatia et al. (2021).

**Figure 4:**
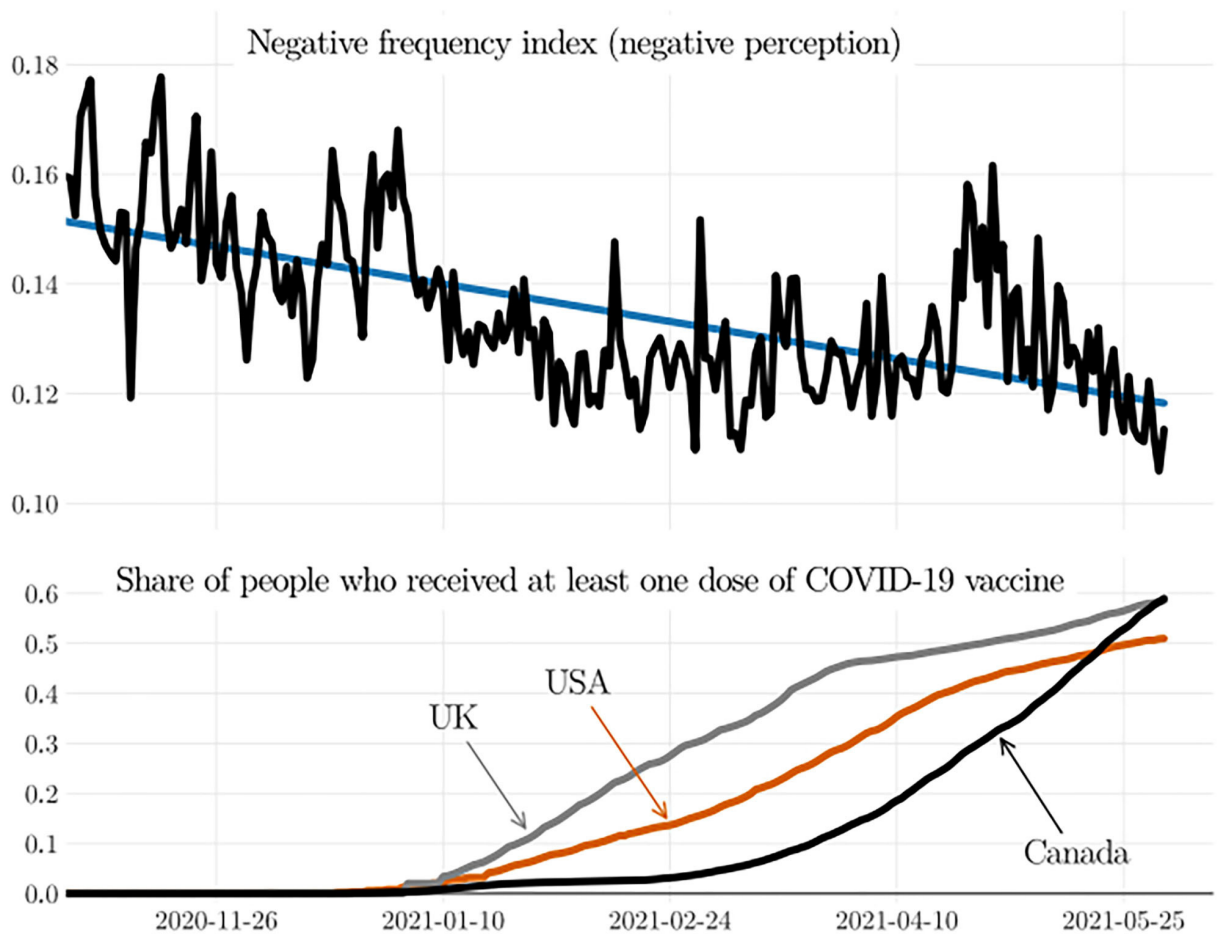
Data from November 01, 2020, to June 01, 2021: negative frequency index (negative perception in tweets) and share of the population in the USA, the UK, and Canada who received at least one dose of the COVID-19 vaccine. The straight line (in blue, upper curve), obtained by linear regression, indicates that the negative perception decreased intensively right after the vaccination in these three countries started.

To explore even further the paradox between the increase of deaths and the decrease of negative perception, we analyzed the vaccination statistics of the USA, the UK, and Canada, see Figure 4. The curves in Figure 4 reveal that there exists a strong relation between dropping negative perception and increased vaccination in these countries. This psychological phenomenon may be caused by the perception of safety among those who got vaccinated, thus confirming that the vaccination has the potential to bring a sense of psychological wellbeing to the population. Note that improving the psychological wellbeing of the people diminishes their chances of developing certain stress-induced diseases (Brosschot, 2010; Wirtz and von Känel, 2017; Marchant et al., 2020).

**Remark 4**. *Data show that the strong decrease in the negativity of the people coincides with the vaccination campaign in the USA, the UK, and Canada (Figure 4). These countries account for most of the tweets. For instance, one study has analyzed the origin of about four million tweets written in English, and it has found that most tweets came from the USA (42.5%), India (10.8%), Canada (5.9%), and the United Kingdom (5.9%), see Garcia and Berton (2021). For this reason, it seems reasonable to infer that vaccination in the USA, the UK, and Canada plays a key role in lowering the negative perception in tweets. Yet, vaccination is not the only cause of this reduced negativity, as discussed in the next section*.

In summary, our findings suggest that the vaccination reduces the negativity of the people, thus improving their wellbeing.

### 3.5. Limitations

This study acknowledges some limitations, as detailed next.

While the data in this study show that the negative perception of the COVID-19 pandemic is dropping, this conclusion cannot be extended to the English-speaking population because our data account for Twitter users only. Generalizing the sentiment of Twitter users for all the population could lead to a bias since millions remain away from social media platforms due to extreme poverty (Steele et al., 2017). For this reason, it is unclear whether our conclusions generalize to the whole English-speaking population.

As for the list of negative words, we recognize that it has a limited vocabulary. Moreover, our counting procedure was blind with respect to misspelling words, a feature that could lead to a loss of potentially important data.

Another limitation of this study is that it neglects the context and semantics in which the negative words appear. By counting the number of negative words in Twitter posts, we overlook irony, sarcasm, metaphor, and other language expressions, key elements for a comprehensive sentiment analysis (Poria et al., 2020; Mohammad, 2021). For instance, although a COVID-related tweet containing the phrase “*living in a hotel is not so bad”* seems to express a positive emotion, our counting method interprets that phrase as negative because the word *bad* is in the list of negative words (see Table 1). Even though recognizing this limitation, we believe that the counting method captures at least a glance of overall negative perception.

In addition to vaccination, it is unclear what factors contribute to diminishing the negative perception toward the pandemic. Perhaps a contributing factor is the reopening of the US economy. In May 2021, the unemployment rate in the USA was at 5.8%, a level slightly above that reached before the start of the pandemic (data from the USA Bureau of Labor Statistics at www.bls.gov, accessed on June 9, 2021). The economy of the UK and Canada followed a similar trend. Diminishing negative perception seems a consequence of the good performance of the economy in these countries—more people working results in less depression, anxiety, and negative feelings (Murphy and Athanasou, 1999).

At present, it is unclear how much of the negative perception in Twitter comes from people who deny COVID and its deadly consequences. Strong on social media, the movement called *science denial* comprises people spreading misinformation and fake news (for further details as to how misinformation flows through Twitter, Facebook, and Youtube, see Cheng et al., 2021; Lavorgna and Myles, 2021; Yang et al., 2021). Misinformation and fake news can lead people to engage in activities that increase their risk of getting or spreading COVID, like refusing to wear masks in public indoor spaces (e.g., Escandón et al., 2021). It is worth noting that people tend to show low adherence to wearing masks (Huynh, 2020b), even though researchers have shown that wearing masks reduce the spread of COVID (e.g., Mitze et al., 2020; Howard et al., 2021). Also effective in containing the spread of COVID is social distancing, a behavior that depends heavily on the local culture (Huynh, 2020a).

Recent data indicate that a large proportion of the population reverberates misinformation and fake news. For instance, using data from the beginning of the pandemic, the authors of Latkin et al. (2021) report that around 8% of Americans had believed that COVID was not worse than the flu, a thought contrary to the scientific evidence that shows that the infection fatality rate of COVID is much worse than that of the flu (Levin et al., 2020). Moreover, using data from July 2020, the author of Cornwall (2020) reports that 25% of Americans were against taking COVID vaccines when available. This information aligns well with a recent study that says that 22% of Americans identify themselves as anti-vaccine activists (Motta et al., 2021). These examples are evidence that the science denial movement finds an echo in society (Bessi et al., 2015).

More recently, a collective has started using Twitter to attack Covid vaccines (Herrera-Peco et al., 2021; Muric et al., 2021). Having studied the contents of COVID-related posts on Twitter, the authors of Germani and Biller-Andorno (2021) discovered that members of the anti-vaccination group write posts with a strong emotional tone. Emotion was dominant in 25% of the posts from the anti-vaccination group, whereas emotion was detected in only 0.3% of the posts from the pro-vaccination group (c.f, Germani and Biller-Andorno, 2021). Moreover, about 20% of vaccine-related posts were written by anti-vaccine activists (Yousefinaghani et al., 2021). We acknowledge that the emotion imprinted in Twitter by the anti-vaccine group may skew the data we used to construct the negative perception of the COVID pandemic.

## 4. Concluding Remarks

This study has shown that the negative perception of the people of the COVID-19 pandemic is dropping. The methodology we have used to reach this conclusion is as follows. First, we have built up a list of negative words, striving to keep only the words that bring a strong negative sentiment. Using around 150 million Twitter posts related to the COVID-19 pandemic, we have calculated the corresponding negative frequency index, which equals the number of negative words divided by the number of posts, and this index is computed on a daily basis. As evidenced by the data, the negative frequency diminishes as long as the pandemic evolves (see Figure 3).

Because the USA corresponds to around 42.5% of all tweets (e.g., Garcia and Berton, 2021), some people might expect that the negativity of the people would increase as the number of deaths in the USA skyrocketed. However, we have observed the opposite—a paradox. Namely, while the number of deaths in the USA had risen steeply, the negativity of the people decreased. This paradox finds support in *psychophysical numbing*, a human trait summarized by some researchers in the quote “*the more who die, the less we care,”* see Slovic and Västfjäll (2015); Dyer and Kolic (2020); Bhatia et al. (2021).

Finally, it is worth mentioning that we see a drastic decrease in the negative perception when the vaccination campaign started in the USA, Canada, and the UK, see Figure 4. Because these three countries account for more than 50% of the Twitter posts (e.g., Garcia and Berton, 2021), we believe that vaccination has played an important role in decreasing the negativity of the people.

## Data Availability Statement

Publicly available datasets were analyzed in this study. This data can be found here: https://github.com/labcontrol-data/covid19_twitter.

## Ethics Statement

This study has received IRB approval from Vanderbilt University (IRB #211232). Regarding the other institutions involved in this study, ethical review and approval was not required for the study on human participants in accordance with the local legislation and institutional requirements. Written informed consent from the participants was not required to participate in this study in accordance with the national legislation and the institutional requirements.

## Author Contributions

All authors listed have made a substantial, direct and intellectual contribution to the work, and approved it for publication.

## Funding

Research supported in part by the Brazilian agency CNPq Grant 421486/2016-3; 305998/2020-0. JMB partially supported by the National Institute of Aging through Stanford University's Stanford Aging and Ethnogeriatrics Transdisciplinary Collaborative Center (SAGE) center (award 3P30AG059307-02S1).

## Conflict of Interest

The authors declare that the research was conducted in the absence of any commercial or financial relationships that could be construed as a potential conflict of interest.

## Publisher's Note

All claims expressed in this article are solely those of the authors and do not necessarily represent those of their affiliated organizations, or those of the publisher, the editors and the reviewers. Any product that may be evaluated in this article, or claim that may be made by its manufacturer, is not guaranteed or endorsed by the publisher.
